# The genome sequence of the dotted bee-fly, *Bombylius discolor* (Mikan, 1796)

**DOI:** 10.12688/wellcomeopenres.18614.1

**Published:** 2022-12-21

**Authors:** Gavin R. Broad, Natural History Museum Genome Acquisition Lab, Darwin Tree of Life Barcoding collective, Wellcome Sanger Institute Tree of Life programme, Wellcome Sanger Institute Scientific Operations: DNA Pipelines collective, Tree of Life Core Informatics collective, Darwin Tree of Life Consortium

**Affiliations:** 1 Department of Life Sciences, Natural History Museum, London, UK

**Keywords:** Bombylius discolor, dotted bee-fly, genome sequence, chromosomal, Diptera

## Abstract

We present a genome assembly from an individual female *Bombylius discolor *(the dotted bee-fly; Arthropoda; Insecta; Diptera; Bombyliidae). The genome sequence is 280 megabases in span. Most of the assembly (99.93%) is scaffolded into six chromosomal pseudomolecules, with the X sex chromosome assembled. The mitochondrial genome has also been assembled and is 16.7 kilobases in length. Genome annotation identified 10,411 protein-coding genes.

## Species taxonomy

Eukaryota; Metazoa; Ecdysozoa; Arthropoda; Hexapoda; Insecta; Pterygota; Neoptera; Endopterygota; Diptera; Brachycera; Muscomorpha; Asiloidea; Bombyliidae; *Bombylius*; *Bombylius discolor* (Mikan, 1796) (NCBI txid:2741128).

## Background

The dotted bee-fly, *Bombylius discolor*, is a charismatic fly of early spring with a mainly southern distribution in England and Wales, up into the Midlands ( *National Biodiversity Atlas (NBN) Atlas*, no date), and it appears to be increasing its range. *B. discolor* resembles the more common *B. major*, but is darker, with spotted wings, and females have a distinctive line of fuzzy white spots down the mid-line of the abdomen ( Stubbs & Drake, 2014). Excellent resources exist for the identification of *B. discolor* and other bombyliids, including Stubbs and Drake, 2014, Steven Falk’s flickr pages and a photo ID guide associated with Bee-fly Watch, a recording initiative in Britain under the auspices of the Soldierflies and Allies Recording Scheme.

This species is widespread across southern and central Europe, and into central Asia. Mainly a species of open ground, *B. discolor* larvae are parasitoids of mining bees of the genus *Andrena*, particularly *A. flavipes* and *A. cineraria* ( Ismay, 1999). Eggs are flicked backwards into the entrances of bee nest burrows, although they are not always accurate and frequently oviposit on non-target substrates ( Boesi *et al.*, 2009). Bee-flies are obligate flower visitors as the females require pollen to mature their eggs.

The genome of *B*. *discolor* was sequenced as part of the Darwin Tree of Life Project, a collaborative effort to sequence all named eukaryotic species in the Atlantic Archipelago of Britain and Ireland.

## Genome sequence report

**Figure 1.**
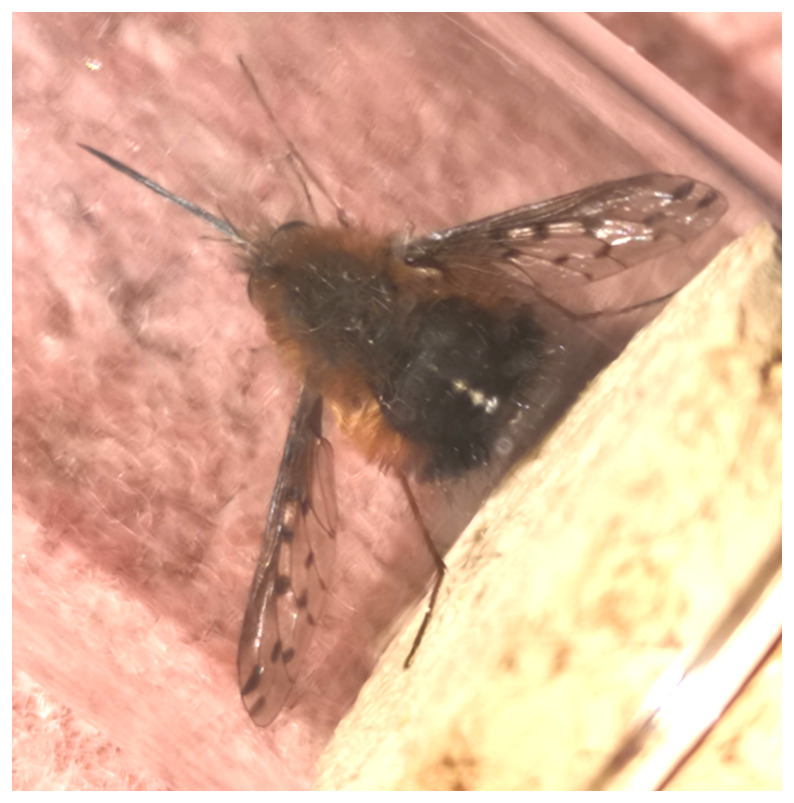
Photograph of the *B. discolor* (idBomDisc1) specimen taken prior to preservation and processing.

The genome was sequenced from an individual female *B. discolor* ( Figure 1) collected from a garden in Tonbridge, Kent, UK. A total of 24-fold coverage in Pacific Biosciences single-molecule HiFi long reads was generated. Primary assembly contigs were scaffolded with chromosome conformation Hi-C data. Manual assembly curation corrected 47 missing/misjoins and removed six haplotypic duplications, reducing the assembly length by 0.07% and the scaffold number by 50% and increasing the scaffold N50 by 31.1%.

**Figure 2.**
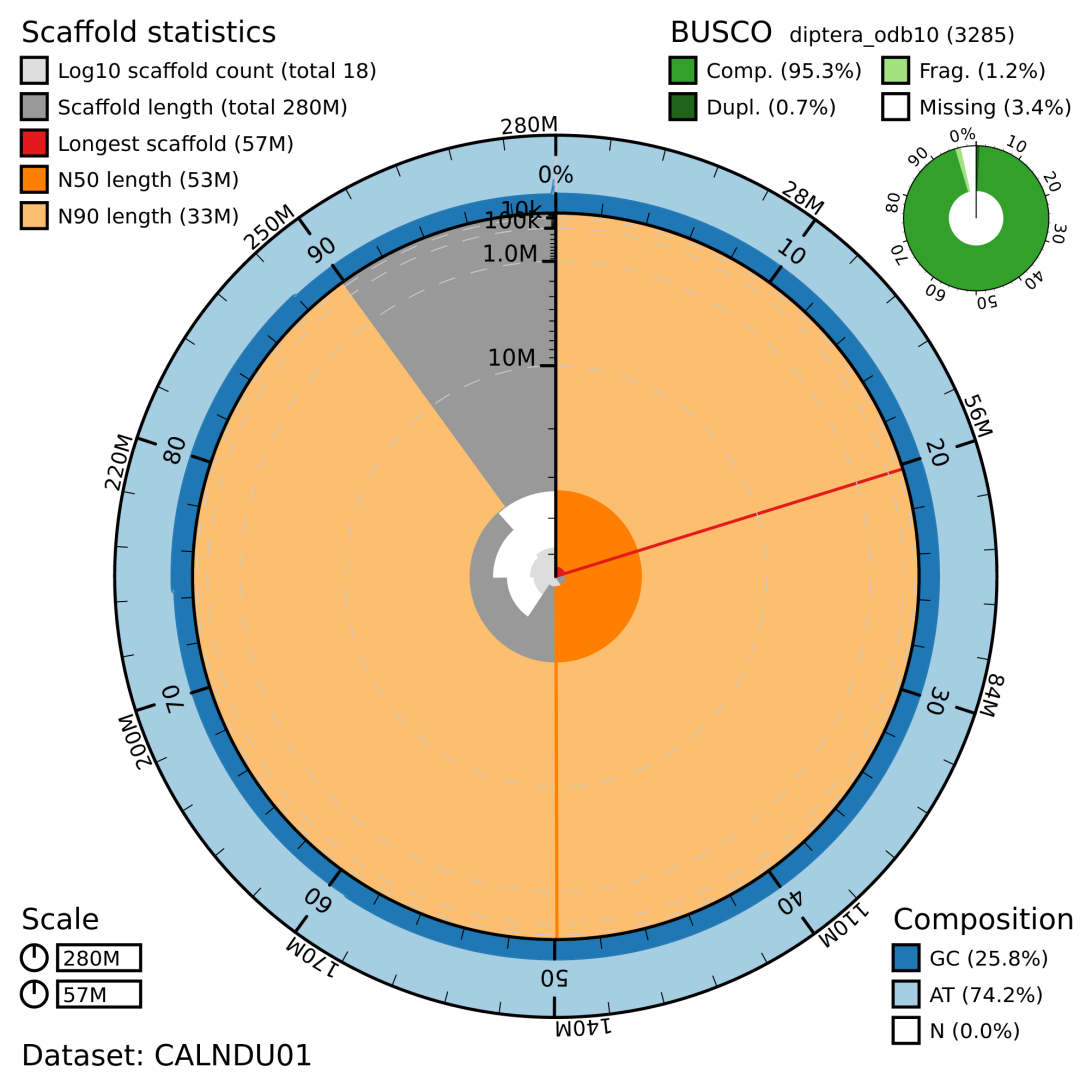
Genome assembly of *B. discolor*, idBomDisc1.1: metrics. The BlobToolKit Snailplot shows N50 metrics and BUSCO gene completeness. The main plot is divided into 1,000 size-ordered bins around the circumference with each bin representing 0.1% of the 279,844,544 bp assembly. The distribution of chromosome lengths is shown in dark grey with the plot radius scaled to the longest chromosome present in the assembly (56,656,967 bp, shown in red). Orange and pale-orange arcs show the N50 and N90 chromosome lengths (53,173,211 and 32,979,702 bp), respectively. The pale grey spiral shows the cumulative chromosome count on a log scale with white scale lines showing successive orders of magnitude. The blue and pale-blue area around the outside of the plot shows the distribution of GC, AT and N percentages in the same bins as the inner plot. A summary of complete, fragmented, duplicated and missing BUSCO genes in the diptera_odb10 set is shown in the top right. An interactive version of this figure is available at https://blobtoolkit.genomehubs.org/view/CALNDU01/dataset/CALNDU01/snail.

The final assembly has a total length of 280 Mb in 17 sequence scaffolds with a scaffold N50 of 53.2 Mb ( Table 1). Most (99.93%) of the assembly sequence was assigned to six chromosomal-level scaffolds, representing five autosomes and the X sex chromosome ( Figure 2– Figure 5; Table 2). The assembly has a BUSCO 5.3.2 ( Manni *et al.*, 2021) completeness of 94.6% using the diptera_odb10 reference set.

**Table 1.** Genome data for *Bombylius discolor*, idBomDisc1.1.

Project accession data	
Assembly identifier	idBomDisc1.1
Species	*Bombylius discolor*
Specimen	idBomDisc1
NCBI taxonomy ID	2741128
BioProject	PRJEB50790
BioSample ID	SAMEA7524252
Isolate information	Female, whole organism
Raw data accessions	
PacificBiosciences SEQUEL I	ERR9527499
Hi-C Illumina	ERR8571692
PolyA RNA-Seq Illumina	ERR10123673
Genome assembly	
Assembly accession	GCA_939192795.1
*Accession of alternate haplotype*	GCA_939192785.1
Span (Mb)	280
Number of contigs	81
Contig N50 length (Mb)	7.4
Number of scaffolds	17
Scaffold N50 length (Mb)	53.2
Longest scaffold (Mb)	56.7
BUSCO * genome score	C:95.3%[S:94.6%,D:0.7%], F:1.2%,M:3.4%,n:3,285
Genome annotation	
Number of protein-coding genes	10,411

* BUSCO scores based on the diptera_odb10 BUSCO set using v5.3.2. C = complete [S = single copy, D = duplicated], F = fragmented, M = missing, n = number of orthologues in comparison. A full set of BUSCO scores is available at https://blobtoolkit.genomehubs.org/view/CALNDU01/dataset/CALNDU01/busco.

**Figure 3.**
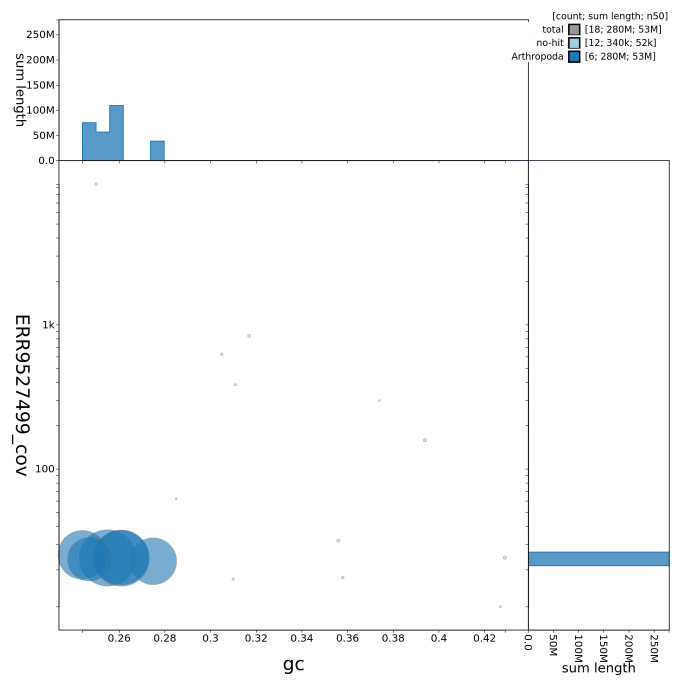
Genome assembly of *B. discolor*, idBomDisc1.1: GC coverage. BlobToolKit GC-coverage plot. Chromosomes are coloured by phylum. Circles are sized in proportion to chromosome length. Histograms show the distribution of chromosome length sum along each axis. An interactive version of this figure is available at https://blobtoolkit.genomehubs.org/view/CALNDU01/dataset/CALNDU01/blob.

**Figure 4.**
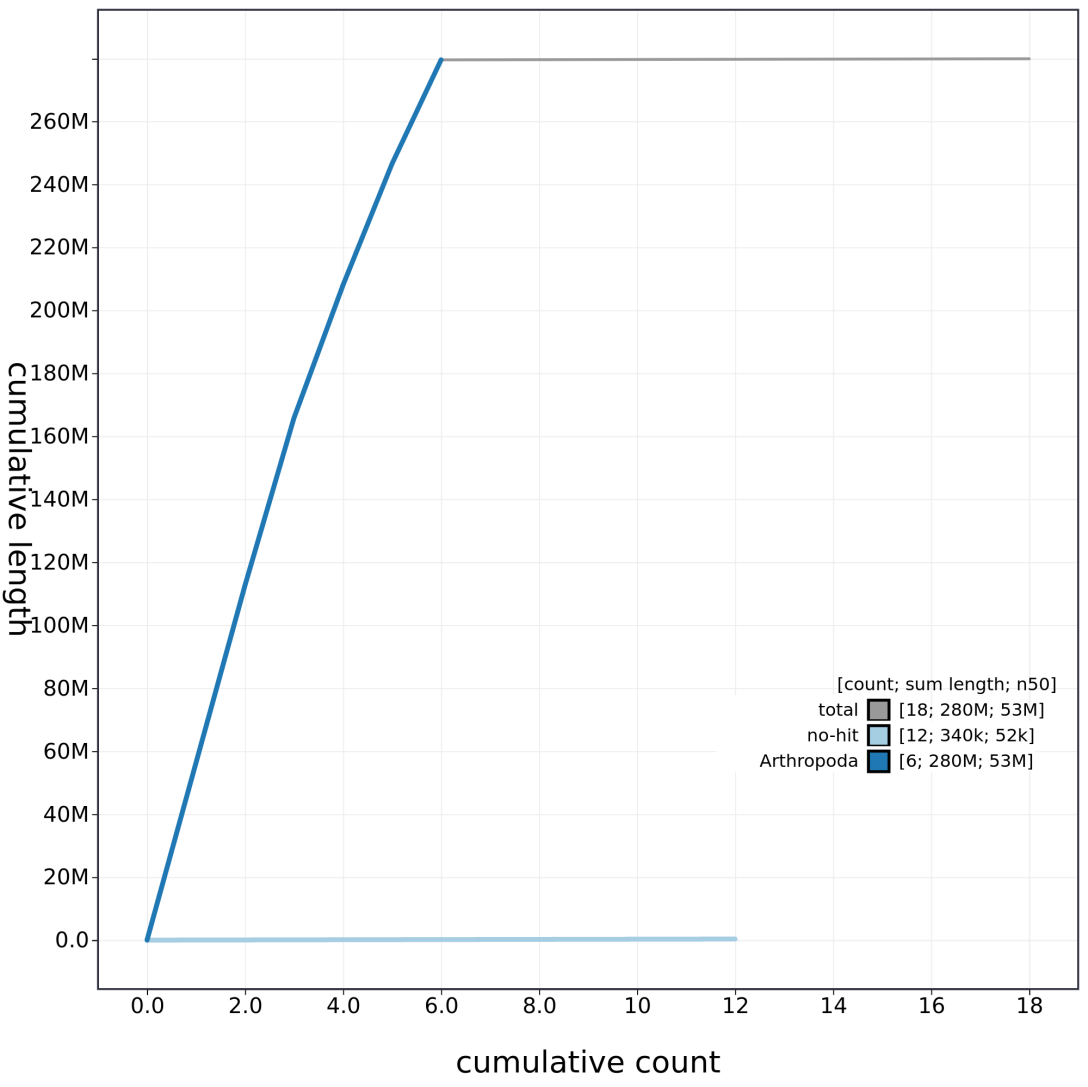
Genome assembly of *B. discolor*, idBomDisc1.1: cumulative sequence. BlobToolKit cumulative sequence plot. The grey line shows cumulative length for all chromosomes. Coloured lines show cumulative lengths of chromosomes assigned to each phylum using the buscogenes taxrule. An interactive version of this figure is available at https://blobtoolkit.genomehubs.org/view/CALNDU01/dataset/CALNDU01/cumulative.

**Figure 5.**
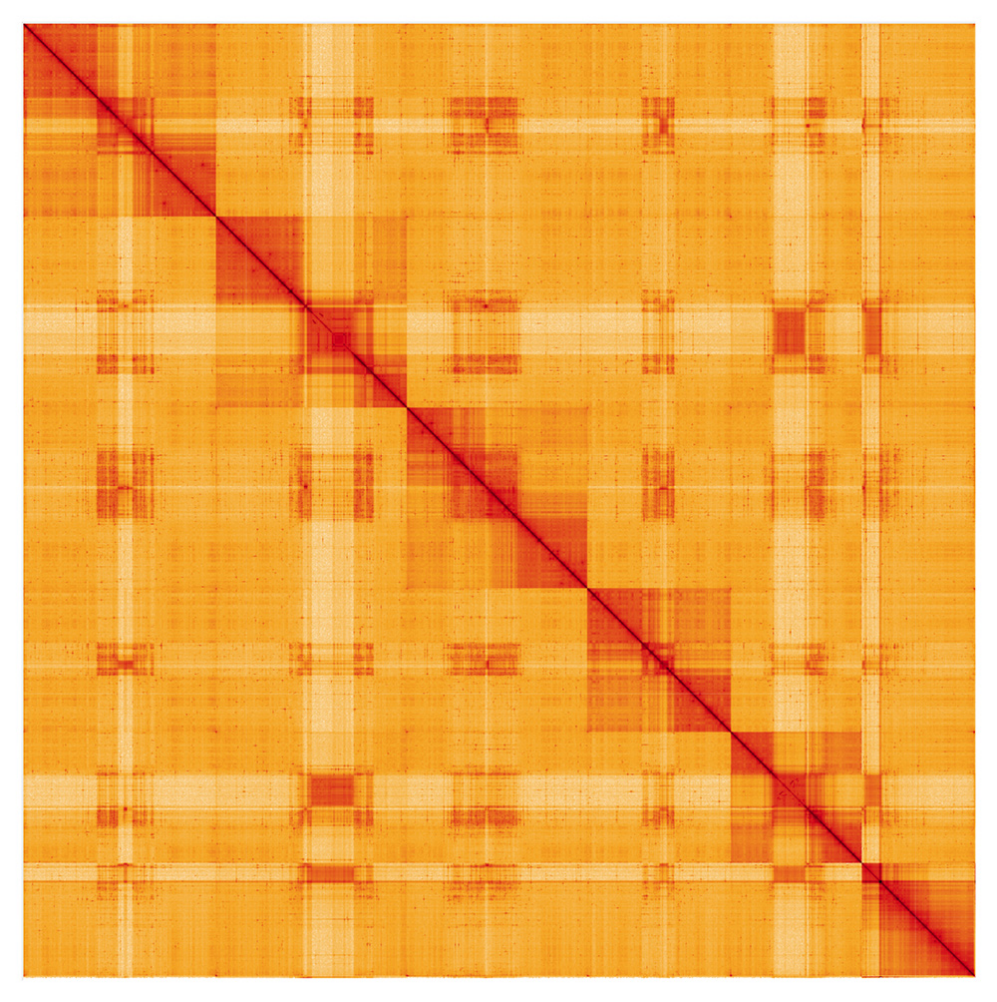
Genome assembly of *B. discolor*, idBomDisc1.1: Hi-C contact map. Hi-C contact map of the idBomDisc1.1 assembly, visualised using HiGlass. Chromosomes are given in order of size from left to right and top to bottom. An interactive view of this plot can be viewed at https://genome-note-higlass.tol.sanger.ac.uk/l/?d=RMaOPk1RTmWaFhZet_PMqQ.

**Table 2.** Chromosomal pseudomolecules in the genome assembly of *B. discolor*, idBomDisc1.

INSDC accession	Chromosome	Size (Mb)	GC%
OW584238.1	1	56.66	25.5
OW584239.1	2	56.04	26.1
OW584240.1	3	53.17	26.1
OW584241.1	4	42.14	24.4
OW584242.1	5	38.52	27.5
OW584243.1	X	32.98	24.7
OW584244.1	MT	0.02	25.1
-	unplaced	0.32	37

While not fully phased, the assembly deposited is of one haplotype. Contigs corresponding to the second haplotype have also been deposited.

## Genome annotation report

The idBomDisc1.1 genome was annotated using the Ensembl rapid annotation pipeline ( Table 1; https://rapid.ensembl.org/Bombylius_discolor_GCA_939192795.1). The resulting annotation includes 16,067 transcribed mRNAs from 10,411 protein-coding and 886 non-coding genes.

## Methods

### Sample acquisition and nucleic acid extraction

A female *B. discolor* (idBomDisc1) ( Figure 1) was collected using a hand net from a garden in Tonbridge, Kent, UK (latitude 51.186304, longitude 0.286534) by Gavin Broad (Natural History Museum), who also identified the species. The sample was preserved by freezing at –80°C.

DNA was extracted from tissue of idBomDisc1 at the Wellcome Sanger Institute (WSI) Scientific Operations core using the Qiagen MagAttract HMW DNA kit, according to the manufacturer’s instructions. Head tissue was set aside for Hi-C sequencing.

DNA was extracted at the Tree of Life laboratory, Wellcome Sanger Institute. The idBomDisc1 sample was weighed and dissected on dry ice with tissue set aside for Hi-C sequencing. Tissue was disrupted using a Nippi Powermasher fitted with a BioMasher pestle. Fragment size analysis of 0.01–0.5 ng of DNA was then performed using an Agilent FemtoPulse. High molecular weight (HMW) DNA was extracted using the Qiagen MagAttract HMW DNA extraction kit. Low molecular weight DNA was removed from a 20 ng aliquot of extracted DNA using 0.8X AMpure XP purification kit. HMW DNA was sheared into an average fragment size of 12–20 kb in a Megaruptor 3 system with speed setting 30. Sheared DNA was purified by solid-phase reversible immobilisation using AMPure PB beads with a 1.8X ratio of beads to sample to remove the shorter fragments and concentrate the DNA sample. The concentration of the sheared and purified DNA was assessed using a Nanodrop spectrophotometer and Qubit Fluorometer and Qubit dsDNA High Sensitivity Assay kit. Fragment size distribution was evaluated by running the sample on the FemtoPulse system. 

RNA was extracted from abdomen tissue of idBomDisc1 in the Tree of Life Laboratory at the WSI using TRIzol, according to the manufacturer’s instructions. RNA was then eluted in 50 μl RNAse-free water and its concentration was assessed using a Nanodrop spectrophotometer and Qubit Fluorometer using the Qubit RNA Broad-Range (BR) Assay kit. Analysis of the integrity of the RNA was done using Agilent RNA 6000 Pico Kit and Eukaryotic Total RNA assay.

### Sequencing

Pacific Biosciences HiFi circular consensus libraries were constructed according to the manufacturers’ instructions. Poly(A) RNA-Seq libraries were constructed using the NEB Ultra II RNA Library Prep kit. DNA and RNA sequencing were performed by the Scientific Operations core at the WSI on Pacific Biosciences SEQUEL II (HiFi) and Illumina NovaSeq 6000 (RNA-Seq) instruments. Hi-C data were generated from head tissue of idBomDisc1 using the Arima v2 kit and sequenced on the Illumina NovaSeq 6000 instrument.

### Genome assembly

The assembly of idBomDisc1.1 is based on 24x PacBio data and Arima2 Hi-C data generated by the Darwin Tree of Life Project ( https://www.darwintreeoflife.org/). The assembly process included the following sequence of steps: initial PacBio assembly generation with Hifiasm ( Cheng *et al.*, 2021), retained haplotig separation with purge_dups ( Guan *et al.*, 2020), and Hi-C based scaffolding with YaHS ( Zhou *et al.*, 2022). The mitochondrial genome was assembled using MitoHiFi ( Uliano-Silva *et al.*, 2021). Finally, the primary assembly was analysed and manually improved using gEVAL ( Chow *et al.*, 2016). Chromosome-scale scaffolds confirmed by the Hi-C data are named in order of size.

The genome was analysed and BUSCO scores were generated within the BlobToolKit environment ( Challis *et al.*, 2020). Table 3 contains a list of all software tool versions used, where appropriate.

**Table 3.** Software tools used.

Software tool	Version	Source
BlobToolKit	3.2.9	( Challis *et al.*, 2020)
gEVAL	N/A	( Chow *et al.*, 2016)
hifiasm	0.16.1-r375	( Cheng *et al.*, 2021)
MitoHiFi	v2	( Uliano-Silva *et al.*, 2021)
purge_dups	1.2.3	( Guan *et al.*, 2020)
YaHS	yahs-1.1.91eebc2	( Zhou *et al.*, 2022)

### Genome annotation

The Ensembl gene annotation system ( Aken *et al.*, 2016) was used to generate annotation for the *B. discolor* assembly (GCA_939192795.1). Annotation was created primarily through alignment of transcriptomic data to the genome, with gap filling via protein-to-genome alignments of a select set of proteins from UniProt ( UniProt Consortium, 2019).

## Data Availability

European Nucleotide Archive: *Bombylius discolor* (dotted bee fly) Accession number PRJEB50790; https://identifiers.org/ena.embl/PRJEB50790. The genome sequence is released openly for reuse. The *Bombylius discolor* genome sequencing initiative is part of the Darwin Tree of Life (DToL) project. All raw sequence data and the assembly have been deposited in INSDC databases. Raw data and assembly accession identifiers are reported in Table 1.

## Author information

Members of the Natural History Museum Genome Acquisition Lab are listed here: https://doi.org/10.5281/zenodo.4790042.

Members of the Darwin Tree of Life Barcoding collective are listed here: https://doi.org/10.5281/zenodo.4893703.

Members of the Wellcome Sanger Institute Tree of Life programme are listed here: https://doi.org/10.5281/zenodo.4783585.

Members of Wellcome Sanger Institute Scientific Operations: DNA Pipelines collective are listed here: https://doi.org/10.5281/zenodo.4790455.

Members of the Tree of Life Core Informatics collective are listed here: https://doi.org/10.5281/zenodo.5013541.

Members of the Darwin Tree of Life Consortium are listed here: https://doi.org/10.5281/zenodo.4783558.
