# Supplementary material for: The genome sequence of the dotted bee-fly, Bombylius discolor (Mikan, 1796)
Source: Wellcome Open Res. 2022 Dec 21;7:306. [Version 1] doi: 10.12688/wellcomeopenres.18614.1 (PMC10133823; doi:10.12688/wellcomeopenres.18614.1)
Supplement: Peer review history

# Peer review: Reviewer response for version 1

**DOI:** 10.21956/wellcomeopenres.20641.r56075

**Published:** 2023-04-26

**Authors:** Lucio Navarro-Escalante

**Affiliations:** 1 Federacion Nacional de Cafeteros de Colombia, Bogotá, Bogota, Colombia

In general it is a well written report with high quality results for the genome assembly.

 Some comments and suggestions:

1. Authors should include any scientific reference (e.g. a paper reference) for the statement: "it appears to be increasing its range". Otherwise this should be removed.
2. Authors should also include a reference for the statement: "Bee-flies are obligate flower visitors as the females require pollen to mature their eggs".
3. I suggest to cite Table 1 at the end of statement "Contigs corresponding to the second haplotype have also been deposited", so the readers can find the accession number of it.
4. I suggest to clarify that gEVAL tool is a genome browser in the Methods. Maybe: "manually improved using gEVAL genome browser".
5. If there was something special in the "select set of proteins from UniProt" in the Methods, then it should be better described here.

Are sufficient details of methods and materials provided to allow replication by others?

Yes

Is the rationale for creating the dataset(s) clearly described?

Yes

Are the datasets clearly presented in a useable and accessible format?

Yes

Are the protocols appropriate and is the work technically sound?

Yes

Reviewer Expertise:

Insect molecular genetics and genomics, plant-insect molecular interactions, insect microbiome analysis

I confirm that I have read this submission and believe that I have an appropriate level of expertise to confirm that it is of an acceptable scientific standard.

# Peer review: Reviewer response for version 1

**DOI:** 10.21956/wellcomeopenres.20641.r54560

**Published:** 2023-02-07

**Authors:** Xuankun Li

**Affiliations:** 1 Department of Biological Sciences, University of Memphis, Memphis, TN, USA

The present paper is one of the series of Darwin Tree of Life genome project, with standardized and clear methods and high-quality results. 

 Some modifications are needed in the Background portion: 

 1. 'it appears to be increasing its range' is there any reference to cite about this claim? One possibility on the increased known distribution is the increasing use of social media. If there isn't any scientific study on the expanding range of this species, please remove this claim. 

 2. "Excellent resources exist for the identification of *B. discolor* and other bombyliids" change to "Excellent resources exist for the identification of *B. discolor* and other **British** bombyliids", remember there are ~5000 species of Bombyliidae found worldwide. Cited references did not cover the fauna out side Britain. 

 3. "Bee-flies are obligate flower visitors as the females require pollen to mature their eggs." change to "Bee-flies are obligate flower visitors, females require pollen to mature their eggs, males visiting flowers as a resource based encounter site but also feed on nectar and pollen ^1^.

Are sufficient details of methods and materials provided to allow replication by others?

Yes

Is the rationale for creating the dataset(s) clearly described?

Yes

Are the datasets clearly presented in a useable and accessible format?

Yes

Are the protocols appropriate and is the work technically sound?

Yes

Reviewer Expertise:

bee fly systematics, HiFi sequencing and genome analysis

I confirm that I have read this submission and believe that I have an appropriate level of expertise to confirm that it is of an acceptable scientific standard.

## References

[1] : The mating system of a bee fly (Diptera: Bombyliidae). I. Non-resource-based hilltop territoriality and a resource-based alternative. *Journal of Insect Behavior* .1990;3(5) : 10.1007/BF01052331 603-617 10.1007/BF01052331
